# The complete mitochondrial genome of *Attacus atlas formosanus* Villiard, 1969 (Lepidoptera: Saturniidae)

**DOI:** 10.1080/23802359.2021.2023333

**Published:** 2022-01-18

**Authors:** Yu-Feng Huang, Hakan Bozdoğan, Tzu-Han Chen, Chia-Chieh Hsieh, Yu-Shin Nai

**Affiliations:** a Department of Entomology, National Chung Hsing University, Taichung City, Taiwan; b Department of computer science and engineering, Yuan-Ze Uinversity, Taoyuan, Taiwan; c Vocational School of Technical Sciences, Department of Plant and Animal Production, Kırşehir Ahi Evran University, Kırşehir, Turkey

**Keywords:** Mitogenome, Saturniidae, Attacini, Attacus atlas formosanus

## Abstract

The complete mitochondrial genome (mitogenome) of *Attacus atlas formosanus* (Villiard, 1969) is 15,280 bp in length, with the typical gene content and arrangement usually observed in Insecta. It contains 13 protein-coding genes, 22 transfer RNA genes, two ribosomal RNA genes, and one AT-rich region. The overall nucleotide composition of the mitogenome was 39.8% A, 12.9% C, 7.7% G, and 39.6% T, with an A + T bias of 79.4%. Phylogenetic analyses of 23 species in Saturniidae and 3 species in Bombycidae by Bayesian inference showed that *A. atlas formosanus* belonged to the Tribe *Attacini*, closely related to Tribe *Saturniini.* Besides, *A. atlas formosanus* is closely related to *A. atlas* with 99% sequence identity. This result well supported the taxonomic position of Saturniidae and their close relationship with the family Bombycidae.

The Atlas moth, *Attacus atlas* (Linnaeus, 1758) (Lepidoptera: Saturniidae), is one of the largest lepidopterans with a wingspan measuring up to 24 cm and a wing surface area of about 160 cm^2^ (Holloway 1987). Moreover, the silks which were produced by *A. atlas* larvae, were alternatively severed for commercial silk production (Reddy et al. 2013). The *Attacus atlas* was first described by Carl Linnaeus under genus *Phalaena* in 1758 and the genus *Attacus* was then established by Linnaeus in 1767. Under the genus *Attacus*, 12 species were listed as valid (Peigler 1983). The population of *A. atlas* (Linnaeus, 1758) was distributed in tropical and subtropical forests located in Java (six subspecies), India (five subspecies), Sumatra (four subspecies), southern China (three subspecies), Sri Lanka (one subspecies), Malaya (one subspecies), Andamans (one subspecies), Simalur (one subspecies), Borneo (one subspecies), Sulawesi (one subspecies), Bali (one subspecies), and four subspecies in Myanmar, Thailand, Laos, and Vietnam (Holloway 1987; Peigler 1983; Roepke 1953). *A. atlas formosanus* (Villiard, 1969) is the largest moth and the endemic subspecies of Taiwan. However, the complete mitochondrial genome (mitogenome) of *A. atlas formosanus* is still being unraveled. Total genomic DNA was extracted from a mixture of nucleopolyhedrovirus (NPV) infected larvae and conducted to DNA sequencing by following the previously published methods (Huang et al. 2019). Reference-assisted *de novo* assembly approach was utilized by using MIRA assembler (Chevreux et al. 2004) with the mitogenome of *A. atlas* isolate SYAU01 (GenBank: NC_021770.1) as the reference genome. Circularity inference was also confirmed as mentioned in previous research (Huang et al. 2015; Jacob Machado et al. 2018) to obtain the complete circular mitogenome. The complete mitogenome of *A. atlas formosanus* is 15,280 bp in size (GenBank: MZ098706). It consisted of 13 protein-coding genes, 22 transfer RNA genes, two ribosomal RNA genes, and one AT-rich region. The overall nucleotide composition of the mitogenome was 39.8% A, 12.9% C, 7.7% G, and 39.6% T, with an A + T bias of 79.4%. Phylogenetic analyses for 23 species in Saturniidae were performed by MrBayes 3.2 (Ronquist et al. 2012), following the analysis procedure in our previous study (Huang et al. 2015). *Papilio maraho*, *Thitarodes renzhiensis* and 3 species in Bombycidae were used as outgroups. The phylogenetic tree showed the relative position that the *A. atlas formosanus* of insular origin is closely related to *A. atlas* of continental origin, and belongs to the Tribe *Attacini* (Figure 1(A)). When these two atlas moths from different geographic origins were compared, it was found that they shared 99% sequence identity with a higher A + T bias (Figure 1(B)). The mitochondrial gene COX1 was used as DNA barcode target for species identification (Chen et al. 2012; Jin et al. 2018; Li et al. 2011; Xu et al. 2019). The start codons for COX1 gene of the lepidopteran insects are not uniform (Hao et al. 2012), which has been extensively discussed and has long remained a matter of some controversy (Kim et al. 2009). *A. atlas formosanus* uses TTG as the start codon while *A. atlas* uses CGA (Chen et al. 2014; Lee et al. 2021; McCullagh et al. 2020). Moreover, a 4-bp TTAG putative initiator of COX1 was observed (Kim et al. 2006; Yukuhiro et al. 2002) but not identified in *A. atlas*. Furthermore, tetranucleotides (Flook et al. 1995) and hexanucleotides (Beard et al. 1993; Mitchell et al. 1993) were also proposed as the start codon for this gene. In addition, the ND4 gene uses ATG as the start codon in *A. atlas* but ATT in *A. atlas formosanus*. The CYTB gene uses ATA as the start codon *A. atlas* but ATG in *A. atlas formosanus*. Though the high mitogenome identity was found between *A. atlas* of continental origin and *A. atlas formosanus* of insular origin, 9 coding gene sequences of *A. atlas formosanus* mitogenome displayed difference from those of *A. atlas* (Figure 1C), presumably because of the geographic evolutionary force that the area between the habitats of *A. atlas* and *A. atlas formosanus* may harbor.

**Figure 1.**
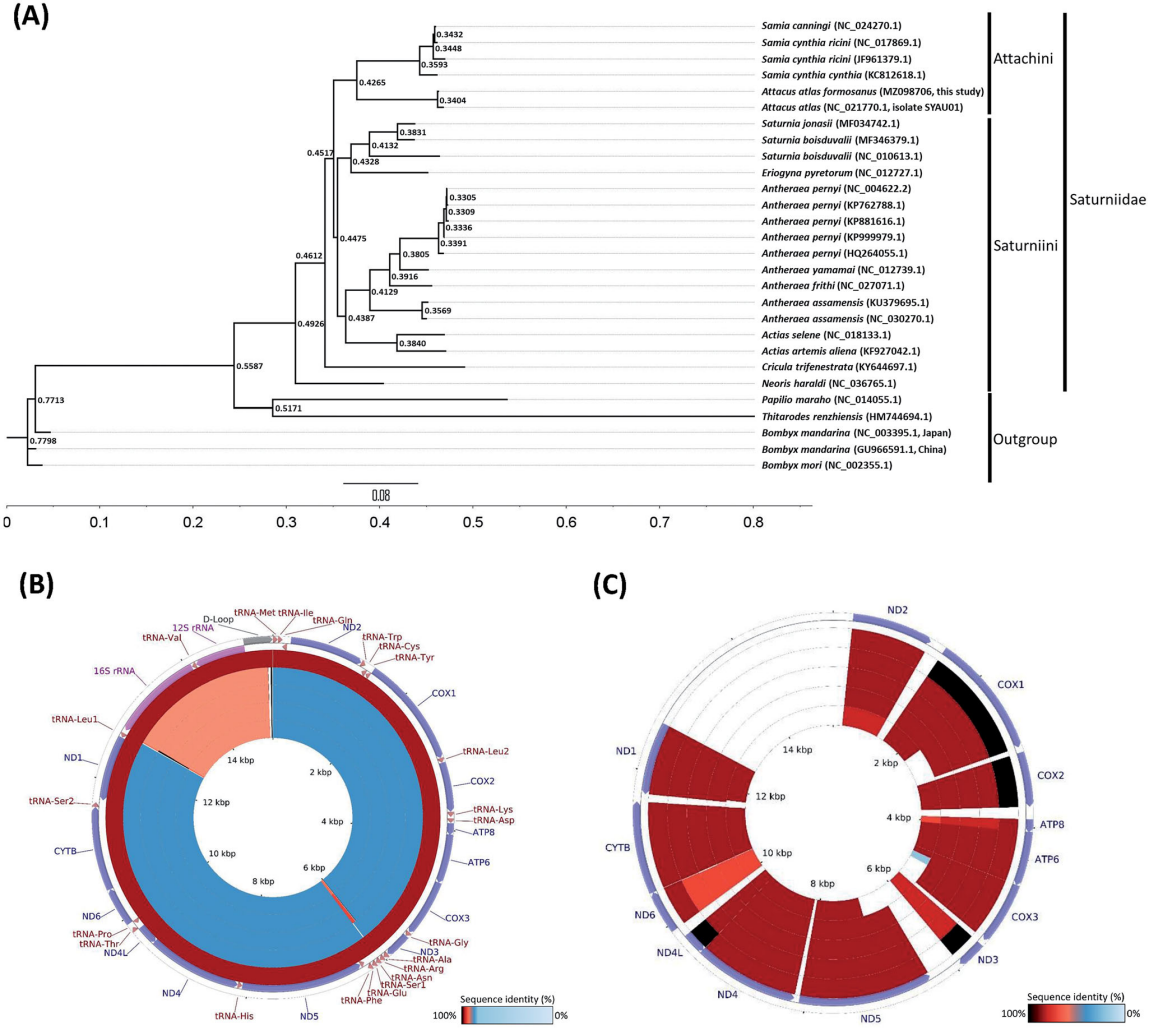
The phylogenetic and comprehensive mitogenomic analysis of mitogenomes from 23 species in Saturniidae. (A) Phylogentic analysis based on whole mitogenomes from 23 species in Saturniidae; *Papilio maraho*, *Thitarodes renzhiensis* and 3 species in Bombycidae are the outgroup; (B) Whole mitogenome comparison; From outer to inner: *Attacus atlas*, *Samia cynthia cynthia*, *Samia canningi*, *Samia cynthia ricini*, and *Samia cynthia ricini*, and (C) Coding gene sequences comparison of 6 closely related species; From outer to inner: *Attacus atlas*, *Samia canningi*, *Samia cynthia cynthia*, *Samia cynthia ricini*, and *Samia cynthia ricini*.

## Data Availability

The mitogenome data is available in NCBI at (https://www.ncbi.nlm.nih.gov), reference number (GenBank: MZ098706).

## References

[CIT0001] Beard CB, Hamm DM, Collins FH. 1993. The mitochondrial genome of the mosquito *Anopheles gambiae*: DNA sequence, genome organization, and comparisons with mitochondrial sequences of other insects. Insect Mol Biol. 2(2):103–124. 9087549 10.1111/j.1365-2583.1993.tb00131.x

[CIT0002] Chen M-M, Li Y, Chen M, Wang H, Li Q, Xia R-X, Zeng C-Y, Li Y-P, Liu Y-Q, Qin L. 2014. Complete mitochondrial genome of the atlas moth, *Attacus atlas* (Lepidoptera: Saturniidae) and the phylogenetic relationship of Saturniidae species. Gene. 545(1):95–101. 24797615 10.1016/j.gene.2014.05.002

[CIT0003] Chen R, Jiang L-Y, Qiao G-X. 2012. The effectiveness of three regions in mitochondrial genome for aphid DNA barcoding: a case in Lachininae. Plos One. 7(10):e46190. 23056258 10.1371/journal.pone.0046190 PMC3463548

[CIT0004] Chevreux B, Pfisterer T, Drescher B, Driesel AJ, Muller WE, Wetter T, Suhai S. 2004. Using the miraEST assembler for reliable and automated mRNA transcript assembly and SNP detection in sequenced ESTs. Genome Res. 14(6):1147–1159. 15140833 10.1101/gr.1917404 PMC419793

[CIT0005] Flook PK, Rowell CH, Gellissen G. 1995. The sequence, organization, and evolution of the Locusta migratoria mitochondrial genome. J Mol Evol. 41(6):928–941. 8587138 10.1007/BF00173173

[CIT0006] Hao J, Sun Q, Zhao H, Sun X, Gai Y, Yang Q. 2012. The complete mitochondrial genome of *Ctenoptilum vasava* (Lepidoptera: Hesperiidae: Pyrginae) and its phylogenetic implication. Comp Funct Genomics. 2012:328049. 22577351 10.1155/2012/328049 PMC3335176

[CIT0007] Holloway JD. 1987. The moths of Borneo, part 3: Lasiocampidae, Eupteroptidae, Bombycidae, Brahmaeidae, Saturniidae, Sphingidae. Kuala Lumpur: Southdene Sdn. Bhd.

[CIT0008] Huang YF, Chen TH, Chang ZT, Wang TC, Lee SJ, Kim JC, Kim JS, Chiu KP, Nai YS. 2019. Genomic sequencing of *Troides aeacus* nucleopolyhedrovirus (TraeNPV) from golden birdwing larvae (*Troides aeacus formosanus*) to reveal defective *Autographa californica* NPV genomic features. BMC Genomics. 20(1):419. 31133070 10.1186/s12864-019-5713-2 PMC6537400

[CIT0009] Huang YF, Midha M, Chen TH, Wang YT, Smith DG, Pei KJ, Chiu KP. 2015. Complete Taiwanese macaque (*Macaca cyclopis*) mitochondrial genome: reference-assisted de novo assembly with multiple k-mer strategy. PLoS One. 10(6):e0130673. 26125617 10.1371/journal.pone.0130673 PMC4488429

[CIT0010] Jacob Machado D, Janies D, Brouwer C, Grant T. 2018. A new strategy to infer circularity applied to four new complete frog mitogenomes. Ecol Evol. 8(8):4011–4018. 29721275 10.1002/ece3.3918 PMC5916287

[CIT0011] Jin Q, Hu X-M, Han H-L, Chen F, Cai W-J, Ruan Q-Q, Liu B, Luo G-J, Wang H, Liu X, et al. 2018. A two-step DNA barcoding approach for delimiting moth species: moths of Dongling Mountain (Beijing, China) as a case study. Sci Rep. 8(1):14256. 30250036 10.1038/s41598-018-32123-9 PMC6155206

[CIT0012] Kim I, Lee EM, Seol KY, Yun EY, Lee YB, Hwang JS, Jin BR. 2006. The mitochondrial genome of the Korean hairstreak, *Coreana raphaelis* (Lepidoptera: Lycaenidae). Insect Mol Biol. 15(2):217–225. 16640732 10.1111/j.1365-2583.2006.00630.x

[CIT0013] Kim MI, Baek JY, Kim MJ, Jeong HC, Kim KG, Bae CH, Han YS, Jin BR, Kim I. 2009. Complete nucleotide sequence and organization of the mitogenome of the red-spotted apollo butterfly, *Parnassius bremeri* (Lepidoptera: Papilionidae) and comparison with other lepidopteran insects. Mol Cells. 28(4):347–363. 19823774 10.1007/s10059-009-0129-5

[CIT0014] Lee KH, Kim MJ, Wang AR, Park JS, Kim SS, Kim I. 2021. Complete mitochondrial genome of *Acanthopsyche nigraplaga* (Lepidoptera: Psychidae). Mitochondrial DNA B Resour. 6(3):1091–1093. 33796751 10.1080/23802359.2021.1899876 PMC7995843

[CIT0015] Li Q-Q, Li D-Y, Ye H, Liu X-F, Shi W, Cao N, Duan Y-Q. 2011. Using COI gene sequence to barcode two morphologically alike species: the cotton bollworm and the oriental tobacco budworm (Lepidoptera: Noctuidae). Mol Biol Rep. 38(8):5107–5113. 21181271 10.1007/s11033-010-0658-1

[CIT0016] McCullagh BS, Alexiuk MR, Payment JE, Hamilton RV, Lalonde MML, Marcus JM. 2020. It’s a moth! It’s a butterfly! It’s the complete mitochondrial genome of the American moth-butterfly *Macrosoma conifera* (Warren, 1897) (Insecta: Lepidoptera: Hedylidae)!. Mitochondrial DNA Part B. 5(3):3615–3617. 10.1080/23802359.2020.1831991 PMC7594742 33367038

[CIT0017] Mitchell SE, Cockburn AF, Seawright JA. 1993. The mitochondrial genome of *Anopheles quadrimaculatus* species A: complete nucleotide sequence and gene organization. Genome. 36(6):1058–1073. 8112570 10.1139/g93-141

[CIT0018] Peigler RS. 1983. A revision of the Indo-Australian genus *Attacus* (Lepidoptera: Saturniidae) [microform]. Texas A&M University. Texas A&M University. Libraries. Available electronically from https://hdl.handle.net/1969.1/DISSERTATIONS-541488.

[CIT0019] Reddy N, Zhao Y, Yang Y. 2013. Structure and properties of cocoons and silk fibers produced by *Attacus atlas*. J Polym Environ. 21(1):16–23.

[CIT0020] Roepke W. 1953. Attacus dohertyi dammermani nov. subspec., and some notes concerning the genus Attacus L.(Lepidoptera Heterocera, Family Saturniidae). Zoologische Mededelingen. 32:49–56.

[CIT0021] Ronquist F, Teslenko M, van der Mark P, Ayres DL, Darling A, Höhna S, Larget B, Liu L, Suchard MA, Huelsenbeck JP. 2012. MrBayes 3.2: efficient Bayesian phylogenetic inference and model choice across a large model space. Syst Biol. 61(3):539–542. 22357727 10.1093/sysbio/sys029 PMC3329765

[CIT0022] Xu Y, Zhang S, Wang H, Wang M, Li G. 2019. Mitochondrial gene sequence (COI) reveals the genetic structure and demographic history of *Lymantria dispar* (Lepidoptera: Erebidae: Lymantriinae) in and around China. Insects. 10(5):146. 10.3390/insects10050146 PMC6572239 31121918

[CIT0023] Yukuhiro K, Sezutsu H, Itoh M, Shimizu K, Banno Y. 2002. significant levels of sequence divergence and gene rearrangements have occurred between the mitochondrial genomes of the wild mulberry silkmoth, *Bombyx mandarina*, and its close relative, the domesticated silkmoth, *Bombyx mori*. Mol Biol Evol. 19(8):1385–1389. 12140251 10.1093/oxfordjournals.molbev.a004200

